# Checkpoint inhibitors synergize with therapeutic platforms, ZVex™ and GLAAS™ by enhancing lentiviral vector-induced tumor-specific immunity and adjuvant-mediated anti-tumor efficacy

**DOI:** 10.1186/2051-1426-3-S2-P346

**Published:** 2015-11-04

**Authors:** Tina C Albershardt, Andrea J Parsons, Jan ter Meulen, Peter Berglund

**Affiliations:** 1 Immune Design, Seattle, WA, USA

## 

The dysregulation of immune checkpoints by tumors is an important mechanism of immune resistance, as administration of checkpoint inhibitors has resulted in impressive clinical responses in patients with late stage cancers. However, a subset of patients exhibits insufficient or no clinical response presumably due to the absence of tumor-specific cytotoxic T lymphocytes (CTLs). This supports the rationale to combine checkpoint inhibitors with therapeutic platforms that generate effector T cells.

We have previously shown that the lentiviral vector platform, ZVex^TM^, generates high levels of tumor antigen-specific CTLs that are critical in mediating protection against tumor challenge in mice. Protection was enhanced by subsequent intramuscular injections of recombinant tumor antigen protein with GLA-SE (formulated glucopyranosyl lipid A, a synthetic TLR4 agonist that is the central component of the GLAAS^TM^ platform) or intratumoral injections of G100 (GLA-SE without protein antigen). ZVex and GLAAS are thus complementary platforms capable of generating tumor-specific immunity through the *in vivo* induction of antigen-specific T cells. Here, we evaluated whether checkpoint blockade could further enhance anti-tumor immunity induced by ZVex and/or GLAAS in mouse tumor models.

In C57BL/6 mice, anti-PD-1 or anti-PD-L1 – but not anti-CTLA4 – enhanced a ZVex/mTRP1-induced CD8 T cell response. To determine whether checkpoint inhibition impacted therapeutic efficacy, B16F10 tumor-bearing mice were immunized with ZVex/mTRP1 followed by weekly administrations of anti-PD-1, anti-PD-L1, or anti-CTLA4. Mirroring the immunogenicity results, ZVex/mTRP1-induced anti-tumor protection was enhanced by the addition of anti-PD-1 or anti-PD-L1, but not anti-CTLA4. This is consistent with previous studies in which we demonstrated that the magnitude of antigen-specific CTL response correlated with the degree of anti-tumor protection. Additionally, compared to either anti-PD-1 or anti-PD-L1 alone, the combined administration of both checkpoint inhibitors best improved anti-tumor efficacy induced by ZVex/mTRP1, or G100, or the combination of ZVex/mTRP1 and GLA-SE/TRP1 protein. As PD-L1 is not the only ligand for PD-1 and PD-1 is not the only receptor for PD-L1, these findings suggest that inhibition of signaling through either PD-1 or PD-L1 alone may not be sufficient to completely block the PD-1/PD-L1 checkpoint.

Taken together, combining checkpoint inhibitors with ZVex and GLAAS capitalizes on the strength of each therapeutic platform. ZVex and GLAAS efficiently generate the effector T cells needed for an effective anti-tumor response. By blocking active immune checkpoints, checkpoint inhibitors further enhance ZVex- and/or GLAAS-induced anti-tumor immunity. Our findings support the combination of checkpoint inhibitors with ZVex and/or GLAAS in clinical trials.

